# Coumarins as Potential Antioxidant Agents Complemented with Suggested Mechanisms and Approved by Molecular Modeling Studies

**DOI:** 10.3390/molecules21020135

**Published:** 2016-01-23

**Authors:** Yasameen K. Al-Majedy, Dunya L. Al-Duhaidahawi, Khalida F. Al-Azawi, Ahmed A. Al-Amiery, Abdul Amir H. Kadhum, Abu Bakar Mohamad

**Affiliations:** 1 Department of Chemical & Process Engineering, University Kebangsaan Malaysia (UKM), Bangi, Selangor 43000, Malaysia; yasmin.chem79@gmail.com (Y.K.A.); amir@eng.ukm.my (A.A.H.K.); drab@eng.ukm.my (A.B.M.); 2 Department of Pharmaceutical Chemistry, College of Pharmacy, Kufa University, Najaf 31001, Iraq; dunialafta1982@yahoo.com; 3 Branch of Chemistry, Department of Applied Science, University of Technology (UOT), Baghdad 10001, Iraq; Khalidachemistry@gmail.com

**Keywords:** antioxidant activity, coumarin, DPPH, H_2_O_2_

## Abstract

Syntheses of coumarins, which are a structurally interesting antioxidant activity, was done in this article. The modification of 7-hydroxycoumarin by different reaction steps was done to yield target compounds. Molecular structures were characterized by different spectroscopical techniques (Fourier transformation infrared and nuclear magnetic resonance). Antioxidant activities were performed by using various *in vitro* spectrophometric assays against 1,1-diphenyl-2-picrylhydrazyl (DPPH) radical and hydrogen peroxide (H_2_O_2_). All compounds exhibited high efficiency as antioxidants compared to ascorbic acid. The highest efficiency scavenging activity was found for compound **3** (91.0 ± 5.0), followed by compounds **2** and **4** (88.0 ± 2.00; and 87.0 ± 3.00). Ascorbic acid C was used as a standard drug with a percentage inhibition of 91.00 ± 1.5. The mechanism of the synthesized compounds as antioxidants was also studied. Hartree–Fock–based quantum chemical studies have been carried out with the basis set to 3-21G, in order to obtain information about the three-dimensional (3D) geometries, electronic structure, molecular modeling, and electronic levels, namely HOMO (highest occupied molecular orbital) and LUMO (lowest unoccupied molecular orbital), to understand the antioxidant activity for the synthesized compounds.

## 1. Introduction

**Scheme 1:**
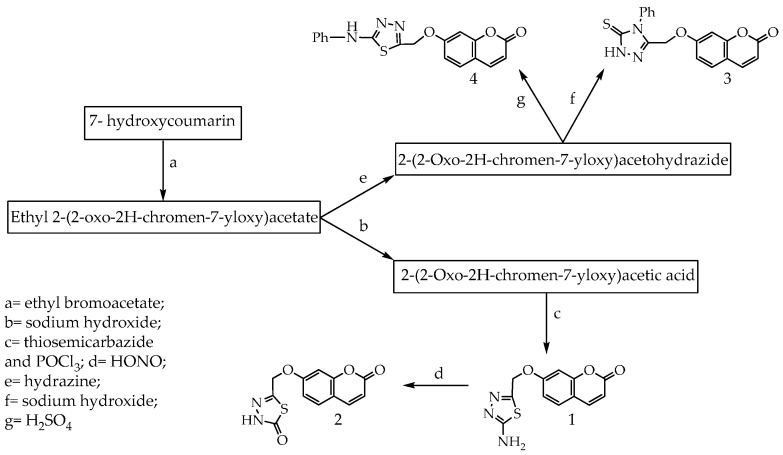
Antioxidant coumarins **1**–**4**.

Coumarins consisting of fused benzene and α-pyrone rings are present in significant amounts in plants, and more than 1300 coumarins have been identified from natural sources [1,2]. Derivatives of coumarins naturally occur as secondary metabolites present in seeds, roots, and leaves of many plant species [3]. Coumarins have a variety of important biological activities such as anti-inflammatory, antioxidant [4,5], antiviral [6], antimicrobial [7] and anti-cancer [8]. Coumarins are indicated to increase central nervous system activity [9,10]. Recently, coumarins have attracted considerable attention for electronic and photonic applications [11,12,13] due to their inherent photochemical characteristics, reasonable stability and solubility in various organic solvents. Many coumarin derivatives have been commercialized as blue-green lasers for fluorescent labels, fluorescent probes [14,15,16] and enzymatic measurements [17]. They exhibit intense fluorescence upon substitution with various functional groups at different positions [18,19]. There is an increasing interest in antioxidants, particularly in those intended to prevent the presumed deleterious effects of free radicals in the human body and to prevent the deterioration of fats and other constituents of foodstuffs. In both cases, there is a preference for antioxidants from natural rather than from synthetic sources [20]. As improved antioxidant status helps to minimize the oxidative damage and thus delay or prevent pathological changes, potential antioxidant therapy should be included either as natural free-radical-scavenging antioxidant enzymes or as an agent which is capable of augmenting the activity of antioxidant enzymes [21]. In a study of scavenging capacity, performed with synthetic coumarins of different substitution patterns, Paya found that only 7,8-dihydroxylated coumarins were active [22]. Hydroxycoumarins are phenolic compounds which act as potent metal chelators and free radical scavengers [23,24,25,26]. To explore the medicinal applications of coumarin derivatives, and in continuation of previous studies [27,28], we focused herein on the design of our approach to increase the antioxidant activity based on a conjugated system and applied theoretical studies to associate the antioxidant activities with electronic structures, with a good relationship between H atom abstraction and unpaired electron delocalization. In order to understand the relationship between the electron delocalization and the reactivity of the radicals, one can examine the electron distribution in the HOMO (highest occupied molecular orbital) and LUMO (lowest unoccupied molecular orbital). The main aim of this work was to optimize structures of all the studied compounds to explain the structure-antioxidant relationship. We had also been concerned with the calculation of antioxidant descriptors: dipole moment, ionization potential (IP), electron affinity (EA), hardness (η), softness (S), and electronegativity (μ) for synthesized coumarins and also the standard compound (ascorbic acid), in addition to determination of the preferred mechanism of antioxidants and the calculation of HOMO and LUMO energies and the band gap. Structure-antioxidant relationships of the synthesized antioxidant and ascorbic acid have been investigated employing the Hartree–Fock–based quantum chemical method together with the 3-21G basis set. Based on the obtained results we conclude that the N-H group is accountable for the antioxidant abilities. Quantum chemical calculations confirmed high antioxidant activity of all synthesized coumarins. Initially we were using 7-hydroxycoumarin as a starting material and all the synthesized coumarins are shown in Scheme 1.

## 2. Results and Discussion

### 2.1. Antioxidant Activity

**Figure 1:**
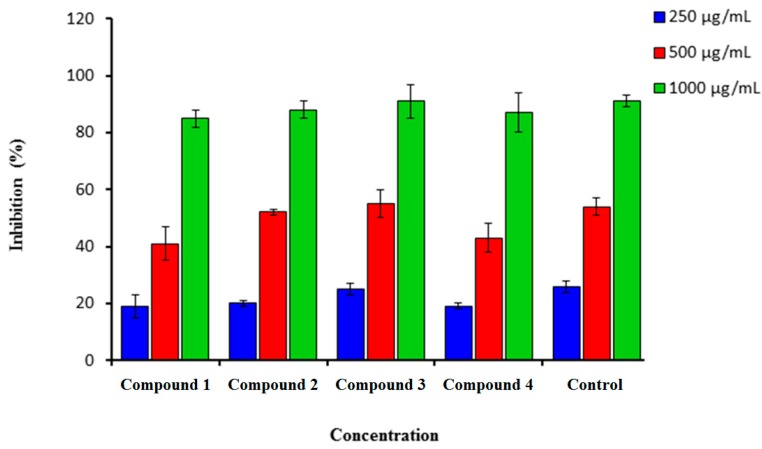
Percentage inhibition of DPPH scavenging activity of synthesized compounds (**1**–**4**) in comparison to Vitamin C. *n* = 3. Error bars indicate standard deviation.

**Figure 2:**
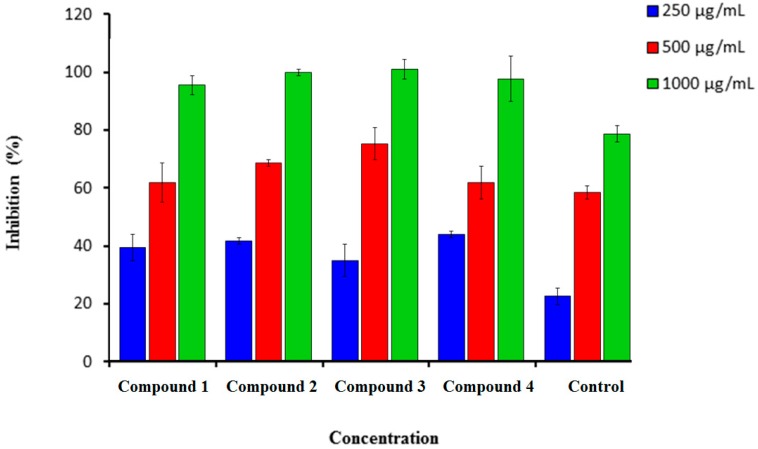
Percentage inhibition of hydrogen peroxide scavenging activity of synthesized compounds (**1**–**4**) in comparison to Vitamin C. *n* = 3. Error bars indicate standard deviation.

Synthesized coumarins **1**–**4** were screened for *in vitro* scavenging activity utilizing DPPH and hydrogen peroxide. These tested coumarins showed high scavenging activity (Figure 1 and Figure 2).

#### 2.1.1. DPPH Scavenging Assay

Figure 1 showed that (**1**–**4**) demonstrated a strong scavenging activity against DPPH at a very low concentration of 250 µg/mL. The highest inhibition for all tested compounds was for the highest concentration which was found at 1000 µg/mL (Figure 1). The highest efficiency scavenging activity was for compound **3** (91.0 ± 5.0), followed by compounds **2** and **4** (88.0 ± 2.00 and 87.0 ± 3.00). Ascorbic acid was used as a standard drug with a percentage inhibition of 91.00 ± 1.5. The hydrogen-donating activity, measured utilizing DPPH as the hydrogen acceptor, demonstrated that a strong association could be found between the concentration of the coumarin molecule and the rate of inhibition [29,30]. Using the hydrogen peroxide test, coumarins **1**–**4** demonstrated their ability to diminish the stable radical.

#### 2.1.2. H_2_O_2_ Scavenging Assay

Hydrogen peroxide can be highly reactive when crossing the cell membrane and form the hydroxyl radical. Figure 2 showed that compounds (**1**–**4**) demonstrated a strong scavenging activity against hydrogen peroxide at a very low concentration of 250 µg/mL, where we observed a concentration-dependent decrease. A very weak inhibitory activity at the lowest concentration (250 µg/mL) was found in compound **3** (42.0 ± 5.00). The highest concentration was found at 1000 µg/mL (Figure 2). The best percentage of scavenging activity was shown by compound **3** (90.0 ± 3.0), followed by compound **2** (89.0 ± 1.00). Ascorbic acid was used as a standard drug with a percentage inhibition of 70.00 ± 2.5.

### 2.2. Postulated Mechanisms for Coumarins **1**, **2**, **3** and **4** as Antioxidants

**Figure 3:**
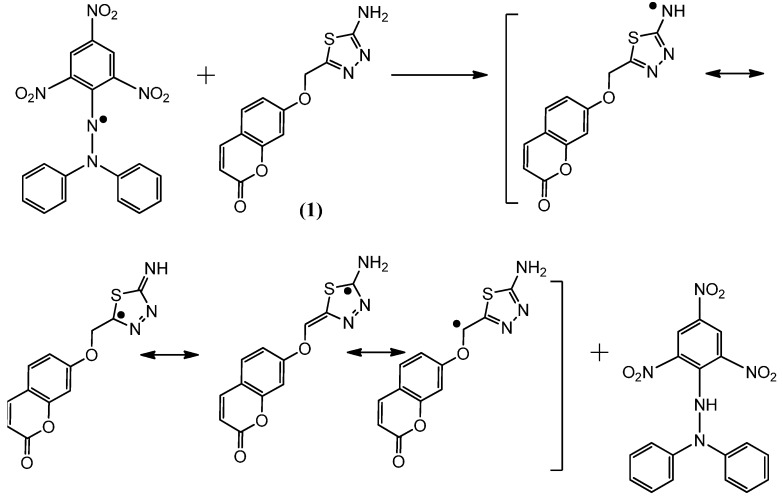
The reaction scheme between DPPH free radicals and compound **1**.

**Figure 4:**
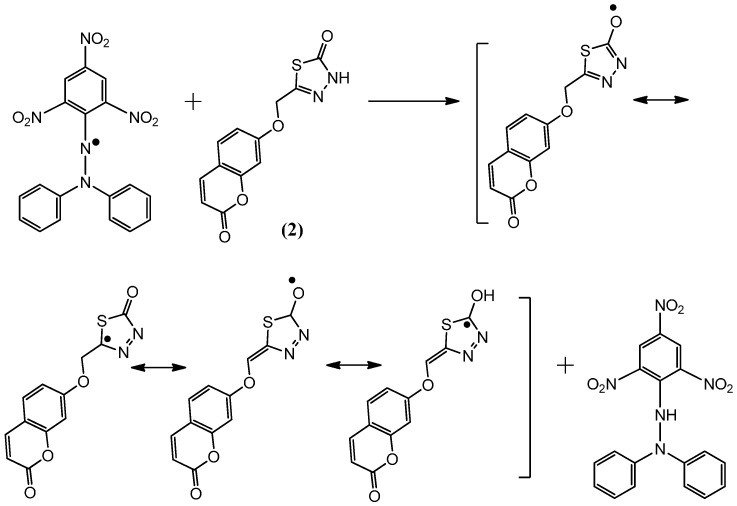
The reaction scheme between DPPH free radicals and compound **2**.

**Figure 5:**
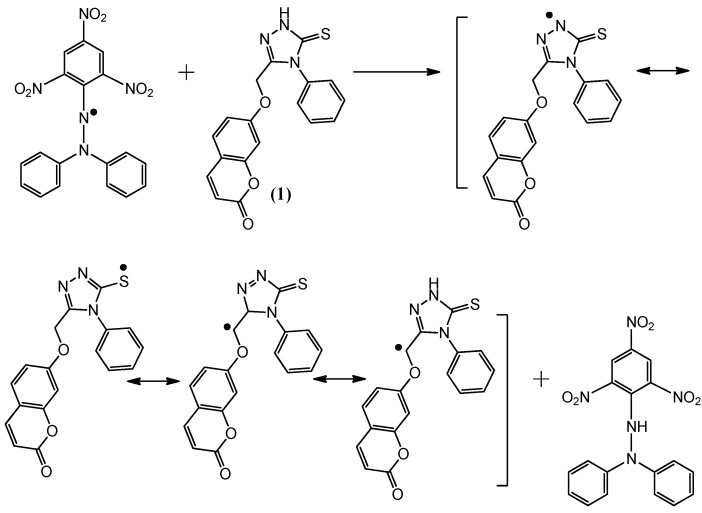
The reaction scheme between DPPH free radicals and compound **3**.

**Figure 6:**
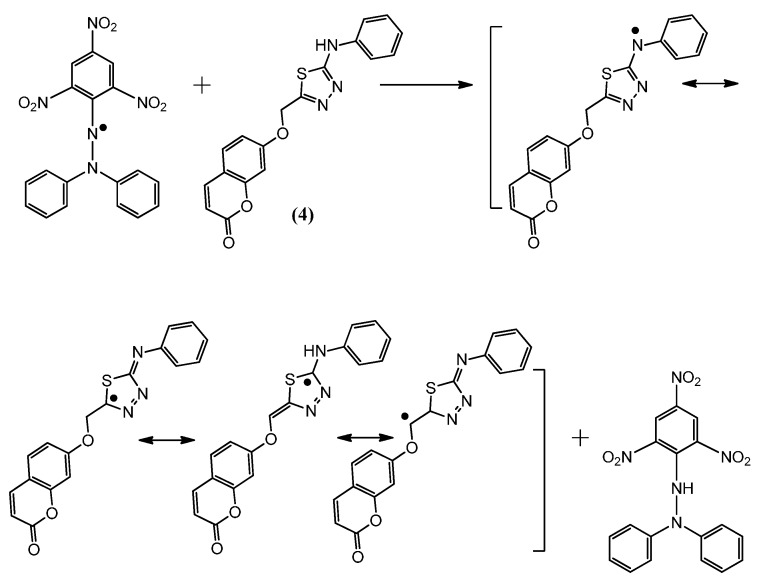
The reaction scheme between DPPH free radicals and compound **4**.

The suggested antioxidant for antioxidant coumarins, as shown in Figure 3, Figure 4, Figure 5 and Figure 6, relies on the hydrogen atoms of the amine group, which were under the influence of resonance and inductive effects. The resonance and inductive effects facilitate the release of hydrogen, resulting in stability of the molecule. Coumarins **1**–**4** have scavenging activities due to the stability of the free radical intermediates of these compounds. An abstraction of a hydrogen atom from the amine group may occur easily [31]. The presence of thiadiazoles, triazole and lactone rings enhances the antioxidant activity. The steric hindrance enhances the antioxidant activity [32,33].

### 2.3. Molecular Modeling Studies

**Figure 7:**
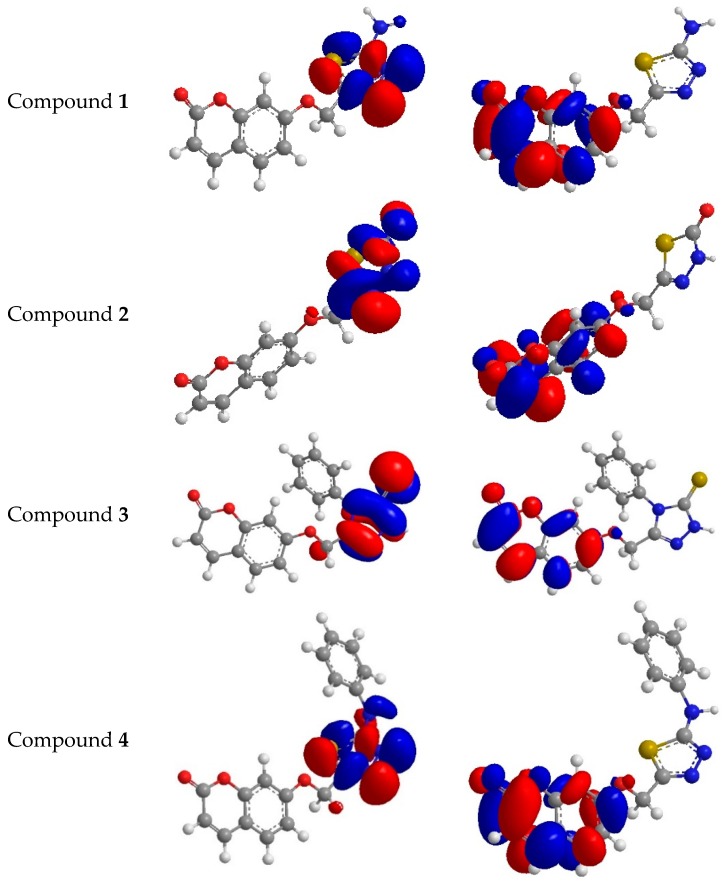
Highest occupied molecular orbital (HOMO) and the lowest unoccupied molecular orbital of compounds **1**–**4**.

To understand the antioxidant activity with the electronic levels, namely HOMO (highest occupied molecular orbital) and LUMO (lowest unoccupied molecular orbital), for antioxidants **1**–**4**, HF (Hartree–Fock)–based quantum chemical studies were carried out with the basis set 3-21G. The energies EHOMO and ELUMO in electron volt values were showed in Figure 7. The compounds with higher antioxidant activity can be confirmed according to the values of EHOMO and ELUMO. In our work we were using the methods of DPPH and peroxide. These methods showed clearly that the scavenging activities of compounds **2** and **3** were higher than those of compounds **1** and **4** and ascorbic acid because of the electron-withdrawing of thionyl, carbonyl and the resonance effect. Theoretically, it was concluded that EHOMO is a good indicator of scavenging activities and the scavenging activities do not depend on ELUMO. The varieties in activities of compounds **1**–**4**, as antioxidants were shown in the calculated EHOMO values, are mostly attributed to pi-electron delocalization, which leads to stability of the free radicals gained after proton abstraction so that pi-electrons delocalized in compounds **1**–**4** also occur in the corresponding radical. The electron density of HOMO could be fully considered to realize the relationship between the delocalizing electrons and the activities of free radicals [34].

Antioxidants **1**–**4** with the highest occupied molecular orbital are delocalized over the whole molecule, which harmonizes the orbital holding unshared electrons. Spin densities of the free radicals that had been created from antioxidants **1**–**4** were compared. High delocalization means the easier creation of free radicals. The spin density appears to be slightly more delocalized for the radicals issued from compounds **2** and **3** than from antioxidants **1** and **4**. The HOMO (highest occupied molecular orbital) energies of target antioxidants **1**–**4**, in addition to the ascorbic acid, are computed as −8.504 eV, −10.102 eV, −8.753 eV, −8.532 eV and −10.772 eV, respectively, while the LUMO energies for target antioxidants **1**–**4** and ascorbic acid are computed as −4.332 eV, −4.132 eV, −4.532 eV, −4.776 eV and −1.115 eV, respectively. The energy gaps for antioxidants **1**–**4** as well as ascorbic acid were respectively as follows: 4.172 eV, 5.790eV, 4.221eV, 3.756eV and 9.655 eV, and this might be due to shifted absorption toward the blue spectrum. The electron delocalizing for thionyl and carbonyl for compounds **2** and **3**, respectively, reveal the variation between HOMO and LUMO of antioxidants **1**–**4**. The comparison of potential for antioxidants **1**–**4** and ascorbic acid as antioxidants according to the band gaps and showed that the highest band gap was for ascorbic acid (control) and then compounds **2** and **3**, and this is highly compatible with experimental results seen in Figure 1 and Figure 2. Dipole moment values of antioxidants **1**–**4** in addition to ascorbic acid demonstrate that all of them are polar molecules and are soluble in polar solvents. IP (ionization potential) affords the understanding of initial energy for releasing an electron from the molecules [35] which means an inverse relation for the antioxidant and IP (Equation (1)).

(1) $$\text{IP}=-E_{\text{HOMO}}$$

EA (electron affinity) is the amount of energy launched when an electron is absorbed by a molecule (Equation (2)). Higher EA leads to easily absorbed electrons, in other words a positive relation with the antioxidant.

(2) $$\text{EA}=-E_{\text{LUMO}}$$

The η (hardness) is charge transfer resistance and S (softness) is the measure of the capacity of an atom to receive electron (Equations (3) and (4)).

(3) $$\eta =-\frac{1}{2}(E_{\text{HOMO}}-E_{\text{LUMO}})$$

(4) $$S=-\frac{2}{\left(E_{\text{HOMO}}-E_{\text{LUMO}}\right)}$$

The µ (electronegativity) is defined as the capacity to attract electrons (Equation (5)) in the chemical bond:

(5) $$µ=-\frac{1}{2}(E_{\text{HOMO}}+E_{\text{LUMO}})$$

Table 1 describes the potential values of the above parameters. These parameters can be supported by the good antioxidant potential. The experimental and calculated theoretical parameters were compared with each other. The calculated data were compared with the experimental values using HF with the basis set of 3-21G. The correlation between experimental and calculated data was found to be good. In addition, HOMO and LUMO analysis of the title molecule were calculated using corresponding methods with the 3-21G basis set. The calculated HOMO-LUMO energies were used to calculate some properties of the title molecule.

**Table 1:** Electronic properties of antioxidants **1**–**4** were obtained by using HF method with the 3-21G basis set.

Parameters	Compound 1	Compound 2	Compound 4	Compound 3	Ascorbic Acid
Dipole moment Depy	4.665	7.117	6.613	5.229	9.549
Ionization potential (IP) eV	8.504	10.102	8.753	8.532	10.772
Electron affinity (EA) eV	4.332	4.132	4.532	4.776	1.115
Hardness (η)	2.86	2.895	2.110	1.378	4.827
Softness (S)	0.239	0.172	0.236	0.266	0.207
Electro negativity(µ)	6.418	7.117	6.142	6.518	5.9435
EHOMO	−8.504	−10.102	−8.753	−8.532	−10.772
ELUMO	−4.332	−4.132	−4.532	−4.776	−1.115
Band gap = E_HOMO_ − E_LUMO_	4.172	5.790	4.221	3.756	9.655

## 3. Materials and Methods

### 3.1. Synthesis of Compounds **1**–**4**

Compounds (**1**–**4**), namely 7-((5-amino-1,3,4-thiadiazol-2-yl)methoxy)coumarin; 5-(((coumarin-7-yl)oxy)methyl)-1,3,4-thiadiazol-2(3*H*)-one; 7-((4-phenyl-5-thioxo-4,5-dihydro-1*H*-1,2,4-triazol-3-yl)methoxy)coumarin and 7-((5-(phenylamino)-1,3,4-thiadiazol-2-yl)methoxy)coumarine, were synthesized according to Al-Amiery 2014 [36].

### 3.2. Antioxidant Activity

#### 3.2.1. DPPH Free Radical Scavenging Activity

The antioxidant activity of synthesis compounds and the standard was assessed on the basis of the radical scavenging effect of the Table 1, 1-diphenyl-2-picrylhydrazyl (DPPH) free radical activity by modified method [37]. The diluted working solutions of the test compound were prepared in methanol. Ascorbic acid was used as standard in 1–100 µg/mL solution. Then 0.002% of DPPH was prepared in methanol and 1 mL of this solution was mixed with 1 mL of sample solution and standard solution separately. These solution mixtures were kept in dark for 30 min and optical density was measured at 517 nm using spectrophotometer. Methanol (1 mL) with DPPH solution (0.002%, 1 mL) was used as blank [38]. The optical density was recorded and % inhibition was calculated using the formula given in Equation (6):

(6) $${\text{DPPH}}_{\text{scavenginig effect }}\text{\%}=A^{{}^{\circ}}-\frac{A}{A^{{}^{\circ}}}\times 100$$

where *A^o^* is the absorbance of the control reaction and 𝐴 is the absorbance in the presence of the samples or standards.

#### 3.2.2. Hydrogen Peroxide Scavenging Activity

A solution of hydrogen peroxide (40 mM) was prepared in phosphate buffer (pH 7.4). Different concentrations (250, 500, and 1000 μg/mL) of synthesized compounds (or ascorbic acid as control) were added to a hydrogen peroxide solution (0.6 mL, 40 mM). Absorbance of hydrogen peroxide at 230 nm was determined after 10 min against a blank solution containing phosphate buffer without hydrogen peroxide [39,40]. Hydrogen peroxide percentage scavenging activity was then calculated using Equation (7):

(7) $$H_{2}O_{2\text{ scavenginig effect }}\text{\%}=A^{{}^{\circ}}-\frac{A}{A^{{}^{\circ}}}\times 100$$

where *A^o^* is the absorbance of the control reaction and 𝐴 is the absorbance in the presence of the samples or standards.

### 3.3. Quantum Studies

The molecular representation sketch of the reference compound was plotted using ChemBioOffice 2010 software. All the quantum chemical calculations were performed using the HF methodology with 3–21G basis set.

### 3.4. Statistical Analysis

The results were expressed as mean ± standard deviation and the statistical significance of differences were determined utilizing one-way analysis of variance (ANOVA) using the SPSS 17.0 statistical software program. Differences were considered significant at *p* < 0.05. The values are presented as mean ± SD (*n* = 3).

## 4. Conclusions

Coumarins were successfully synthesized and characterized by using spectroscopic techniques (FT-IR and NMR). Antioxidant activities were evaluated by DPPH and hydrogen peroxide assays and the results indicated that they have good scavenging activities. Compounds **3** and **2** were found to be an inhibitor of the DPPH and H_2_O_2_ activities with levels of 91.0 ± 5.0, and 88.0 ± 2.00, respectively. Compound **3** was better than the standard reference drug used, and these results were confirmed by modeling studies. The mechanism of the synthesized compounds as antioxidants was also studied. Hartree–Fock–based quantum chemical studies with the basis set 3-21G and molecular modeling were carried out for the synthesized compounds to understand the antioxidant activity. The electronic levels, namely HOMO (highest occupied molecular orbital) and LUMO (lowest unoccupied molecular orbital), were also studied. We had also been concerned with the calculation of antioxidant descriptors: dipole moment, ionization potential (IP), electron affinity (EA), hardness (η), softness (S), and electronegativity (µ) for synthesized coumarins and also the standard compound (ascorbic acid).
